# Old Friends, immunoregulation, and stress resilience

**DOI:** 10.1007/s00424-018-2228-7

**Published:** 2018-11-01

**Authors:** Dominik Langgartner, Christopher A. Lowry, Stefan O. Reber

**Affiliations:** 1 Laboratory for Molecular Psychosomatics, Department of Psychosomatic Medicine and Psychotherapy, University Ulm, Albert-Einstein-Allee 23, 89081 Ulm, Germany; 2 Department of Integrative Physiology and Center for Neuroscience, University of Colorado Boulder, Boulder, CO 80309 USA; 3 Department of Physical Medicine & Rehabilitation and Center for Neuroscience, University of Colorado Anschutz Medical Campus, Aurora, CO 80045 USA; 4 Veterans Health Administration, Rocky Mountain Mental Illness Research Education and Clinical Center (MIRECC), Denver Veterans Affairs Medical Center (VAMC), Denver, CO 80220 USA; 5 Military and Veteran Microbiome Consortium for Research and Education (MVM-CoRE), Denver, CO 80220 USA

**Keywords:** Trier Social Stress Test (TSST), Urban versus rural, Inflammation, Interleukin (IL)-6, Cortisol, Old Friends

## Abstract

There is a considerable body of evidence indicating that chronic adverse experience, especially chronic psychosocial stress/trauma, represents a major risk factor for the development of many somatic and affective disorders, including inflammatory bowel disease (IBD) and posttraumatic stress disorder (PTSD). However, the mechanisms underlying the development of chronic stress-associated disorders are still in large part unknown, and current treatment and prevention strategies lack efficacy and reliability. A greater understanding of mechanisms involved in the development and persistence of chronic stress-induced disorders may lead to novel approaches to prevention and treatment of these disorders. In this review, we provide evidence indicating that increases in immune (re-)activity and inflammation, potentially promoted by a reduced exposure to immunoregulatory microorganisms (“Old Friends”) in today’s modern society, may be causal factors in mediating the vulnerability to development and persistence of stress-related pathologies. Moreover, we discuss strategies to increase immunoregulatory processes and attenuate inflammation, as for instance contact with immunoregulatory Old Friends, which appears to be a promising strategy to promote stress resilience and to prevent/treat chronic stress-related disorders.

## Introduction

Chronic psychosocial stress/trauma is a major burden of modern life and poses a clear risk factor for a plethora of stress-related somatic and affective disorders [213]. Although the prevalence of stress-related somatic and affective disorders has increased over the past decades, the factors contributing to these increases, especially for stress-associated mental disorders, are far from being fully understood. Currently available pharmacologic approaches to treatment of stress-associated mental disorders, such as major depressive disorder, suffer from important shortcomings, including limited efficacy, delayed onset of action, increased relapse risk upon withdrawal, and significant side effects that impair quality of life and promote treatment non-adherence and/or discontinuation [13, 131, 229, 231, 263, 313, 355]. In addition to these shortcomings in treatment of mental health disorders, there is a recognized need to develop strategies for their prevention. Thomas Insel, the former head of the National Institute of Mental Health (NIMH) commented over a decade ago that, “In contrast to researchers in cancer and heart disease who have sought cures and preventions, biological psychiatrists in both academia and industry have set their sights on incremental and marketable advances, such as drugs with fewer adverse effects” [177].

In this review, we focus on increases in inflammation as a potential causal factor in the increased prevalence of stress-related somatic and affective disorders, as proposed by the “Cytokine Theory of Mental Disorders [58, 79, 82, 84],” and reduced exposures to immunoregulatory microorganisms as a factor contributing to increases in chronic low-grade inflammation, as proposed by the “biodiversity” hypothesis [153], “missing microbes” hypothesis [39], or “Old Friends” hypothesis [238, 351]. Briefly, not only many stress-associated somatic disorders, but, interestingly, also mental disorders, are associated with, and at least in part also promoted by, an activated immune status and chronic low-grade inflammation [345]. As these disorders are often further  characterized by a compromised regulatory T (Treg) cell compartment [230, 373], a failure of immunoregulation might, therefore, be involved in promoting an over-reacting of the inflammatory stress response and, thus, predisposing an individual to the development of certain stress-related somatic and mental disorders. The failure of immunoregulation, in turn, is due in part to a lack of exposure to immunoregulatory microorganisms with which humans co-evolved. Thus, interventions that increase immunoregulation and attenuate chronic low-grade inflammation, i.e., contact with immunoregulatory Old Friends, might provide a novel and promising strategy to prevent stress-induced immune activation and to promote stress resilience.

## Stress-associated disorders are on the rise

Chronic stress, particularly, chronic psychosocial stress, poses an acknowledged risk factor for numerous disorders, including: somatic disorders, like cardiovascular diseases [51, 52, 100, 200, 264, 395]; fibromyalgia [9]; bronchial asthma [427, 428]; atopic dermatitis [54]; arthritis [66, 162]; inflammatory bowel disease (IBD) [34, 35, 37, 102, 226, 266, 267, 343, 358]; stomach ulcers [66]; diarrhea and digestive problems [56, 66]; chronic pelvic and abdominal pain [56, 66]; infections [56, 65, 66, 194]; headaches [56, 66]; impaired wound healing [195, 196, 258]; cancerogenesis [193, 225, 338]; as well as affective disorders [342], like major depressive disorder [1, 71, 72, 88, 152, 159, 168, 411, 412], anxiety disorders; and trauma- and stressor-related disorders, such as posttraumatic stress disorder (PTSD) and chronic fatigue syndrome [158]. While the underlying etiologies of these diseases are not fully understood, epidemiologic data provide strong evidence of a steady rise in the incidence of many stress-associated disorders, including allergic diseases, such as allergic asthma [2], atopic dermatitis [157] and hay fever/rhinitis [397], and autoimmune diseases, like multiple sclerosis [135, 324], type 1 diabetes [147], and Crohn’s disease (CD) [116], in developed countries since the 1950s [17]. A recent meta-analysis reported rising incidence rates of Crohn’s disease and ulcerative colitis (UC), the principal types of inflammatory bowel diseases (IBD), in newly industrialized countries in Africa, Asia, and South America, including Brazil and Taiwan, and stably high incidence rates in North America and Europe since 1990 [298], suggesting that the degree of industrialization is positively associated with higher incidence rates of stress- and inflammation-associated disorders, at least until a certain plateau phase is reached. For comparison, in 2015 and 2016, an estimated 3.1 million (unadjusted lifetime prevalence of 1.3%) adults in the USA had at some time received a diagnosis of IBD [430]. Moreover, while Murray and Lopez in 1996 ranked typical stress-associated disease entities like cardiovascular disorders and depression as fifth and fourth, respectively, among the ten leading causes of disability-adjusted life years (DALYs) for the year 1990, their projected rank for these disorders for the year 2020 was first and second, respectively [291]. These estimations are supported by a study published in 2014 reporting that depression is responsible worldwide for more “years lost to disability (YLDs)” than any other condition, due to its high prevalence and the fact that it lasts for many years [182]. The increasing individual and socioeconomic burden of mental disorders is indicated by the fact that the overall “days out of role per year,” i.e., days in the past year, each respondent reported being totally unable to work or carry out their other normal daily activities, due to any mental disorders outnumbered those due to any physical disorders by about 30% in 24 countries that participated in the World Health Organization (WHO) World Mental Health (WMH) surveys [10].

In summary, there is significant demand and significant unmet need for both treatment and prevention of stress-associated somatic pathologies as well as stress-related anxiety and affective disorders. However, promising strategies have not yet been delineated.

## Stress, inflammation, and mental health: the cytokine theory of affective disorders

### Affective disorders are paralleled by increased immune system (re)activity

Many, if not all, of the above referenced stress-associated somatic [7, 50, 69, 234, 314, 323, 357] and psychiatric disorders, including PTSD, generalized anxiety disorder (GAD), panic disorder (PD), phobias (agoraphobia, social phobia, etc.) [142, 277], depression [172, 251, 309], burnout [146, 415], and chronic fatigue syndrome [44, 346, 384], are accompanied by an over-(re)active immune system and chronic low-grade inflammation.

#### Posttraumatic stress disorder

Trauma- and stressor-related disorders, such as PTSD, are associated with chronic low-grade inflammation. Emerging evidence even suggests that inflammation plays a role in vulnerability to PTSD, as well as persistence of PTSD symptoms. For example, women with childhood abuse-related PTSD display increased NF-κB pathway activity, which is positively correlated with PTSD symptom severity, and decreased whole blood monocyte glucocorticoid (GC) sensitivity compared to healthy controls [310]. Moreover, peripheral blood mononuclear cells (PBMCs) from individuals with a diagnosis of PTSD show an increased spontaneous and lipopolysaccharide (LPS)-induced in vitro secretion of proinflammatory cytokines such as interleukin (IL)-6 and IL-1β, when compared to healthy controls [142]. In line with these findings, military combat-related PTSD in male soldiers is associated with higher serum levels of proinflammatory cytokines, even after accounting for depression and early-life trauma [233]. Study participants with a diagnosis of PTSD also have a high risk of developing autoimmune disorders, relative to healthy controls [303], and have exaggerated symptoms of IBD [55, 303], relative to non-PTSD controls. The association between elevated C-reactive protein (CRP) and PTSD was supported in a large general population study, with increased CRP (> 3 mg/L) found in those with a diagnosis of PTSD, compared to those without [375]. Besides evidence for an association between chronic low-grade inflammation and PTSD, the latter presents as a disorder characterized by decreased 24-h average plasma cortisol concentrations (i.e., hypocortisolemia), enhanced negative feedback sensitivity of the HPA axis [87, 223, 262, 288, 431, 432, 435, 436], and increased acute stress-induced cortisol secretion [87].

#### Generalized anxiety disorder, PD, and phobias

There is some evidence that anxiety disorders, including GAD, PD, and phobias, are associated with chronic low-grade inflammation. Male, but not female, study participants with a current anxiety disorder, including GAD, PD, social phobia, or agoraphobia, show increased plasma CRP concentration when compared with healthy controls, based on the Netherlands Study of Depression and Anxiety [414]. Of note, immune dysregulation is found especially in persons with a late-onset anxiety disorder, suggesting the existence of a specific late-onset anxiety subtype with a distinct etiology [414]. Plasma CRP levels were also increased in children diagnosed with GAD [68]. Moreover, individuals with agoraphobia had significantly higher follow-up levels of CRP and tumor necrosis factor (TNF; a proinflammatory cytokine), as well as lower levels of the cardioprotective marker adiponectin, relative to their non-agoraphobic counterparts [418]. In line with these findings, median peripheral cytokine levels for 18 of 20 different cytokines were elevated in individuals with PD compared to age- and gender-matched healthy controls, and the proportion of participants with six or more detectable levels of the most common proinflammatory cytokines and chemokines (eotaxin, GM-CSF, interferon [IFN]-α, IL-1α, IL-1β, IL-6, IL-8, monocyte chemoattractant protein-1 MCP-1] and MIP-1a) was higher in anxiety patients compared with controls [167]. Plasma TNF and IL-17 concentrations were higher in cell cultures containing activated T cells from those with a diagnosis of GAD compared with healthy individuals, while T helper 1 (Th1) and T helper 2 (Th2) cytokines were lower in the anxious group compared to the control subjects [409].

#### Depression

The association between chronic-low grade inflammation and major depressive disorder has been extensively reviewed [58, 79, 81, 245, 278, 279, 331] and, therefore, we highlight only a few of the major findings in the current review article. Early studies from the 1990s already concluded that the established immune cell profile of depressed patients points towards the existence of a systemic immune activation [248]. In detail, this is indicated by a higher number of leukocytes, neutrophils, monocytes, class II major histocompatibility complex (MHC) HLA-DR, and CD4+CD45RA memory T cells, as well as increased numbers of IL-2 receptor-bearing cells in participants diagnosed with depression versus healthy controls. Individuals with minor and major depression without melancholia further show an increased CD4^+^/CD8^+^ ratio, whereas individuals with major depression with melancholia show an increased number of CD3^+^ T cells, CD 19^+^ B cells, and CD8^+^ cytotoxic T cells. Activation of cellular immunity in major depression is further corroborated by findings of increased plasma and urinary neopterin concentrations, which is an accepted marker of activation of cell-mediated immunity [104, 244]. In another study, Maes and colleagues showed that individuals with melancholic depression in comparison to healthy controls exhibit significantly more IL-1β accumulation in culture supernatants of phytohaemagglutinin (PHA)-stimulated lymphocytes [247], with the soluble IL-2-receptor (sIL-2R) accumulation reflecting the magnitude of the PHA-induced lymphocyte stimulation in healthy controls but not in depressed individuals. Moreover, individuals with major depressive disorder compared to healthy controls exhibit dexamethasone non-suppression of lectin-induced blastogenesis and of IL-1β production. Besides ex vivo IL-1β secretion, plasma IL-6 [309], IL-1β, TNF, and CRP [278] concentration and ex vivo IL-6 production in culture supernatants of mitogen-stimulated peripheral leukocytes [251] are increased in depressed individuals compared with healthy participants. Interestingly, a positive correlation between IL-6 in the supernatants and postdexamethasone cortisol values again suggests development of GC resistance in depressed individuals. Further data supporting cardinal features of an inflammatory response in patients with major depression are reviewed elsewhere [245, 278, 279]. Briefly, these include increased cytokine receptor expression, acute phase reactants, chemokines, and soluble adhesion molecules in peripheral blood and cerebrospinal fluid (CSF), as well as elevated expression of a variety of innate immune genes and proteins, including IL-1β, IL-6, TNF, TLR3, and TLR4, in postmortem brain samples from suicide victims that had depression. Moreover, peripheral blood gene expression profiles are consistent with a proinflammatory “M1” macrophage phenotype, and polymorphisms in the proinflammatory genes IL-1β, TNF, and CRP genes have been associated with depression and its response to treatment.

#### Burnout syndrome

Burnout has been defined as a combination of depersonalization, emotional exhaustion, and reduced personal accomplishment caused by chronic work stress [259]. Although burnout is not included in the 5th edition of the *Diagnostic and Statistical Manual of Mental Disorders* (*DSM-5*) [12], some countries, for example, Sweden, consider burnout syndrome to be a legitimate justification for sick leave [132]. Besides reporting more job strain and less social support at work, as well as higher levels of anxiety, depression, vital exhaustion, and sleep impairments, female participants with high burnout manifest higher levels of plasma TNF, but not the anti-inflammatory cytokine transforming growth factor beta (TGF-β), independent of confounders including depression [146]. In line with these data, burnout was also in another study associated with increased systemic inflammation, indicated by the fact that higher levels of total burnout symptoms predict higher plasma TNF levels [415] and the fact that individuals with burnout syndrome have an increased risk to develop cardiovascular pathologies [273].

#### Chronic fatigue syndrome

Chronic fatigue syndrome is a medical disorder characterized by “physical and mental fatigue exacerbated by physical and mental effort, as well as subjective cognitive impairment, disrupted and unrefreshing sleep, and some degree of widespread pain” [364]. As with burnout syndrome, chronic fatigue syndrome is not included in the *DSM-5*, and there is ongoing debate regarding whether it should be considered as a psychiatric disorder [364]. Longitudinal studies in individuals with stress-related chronic fatigue indicate that a decrease in both GC and catecholamine sensitivity of immune cells with ongoing stress is associated with self-maintaining inflammation and inflammatory disinhibition under acute stress conditions, which, in turn, lead to fatigue [384]. In detail, increased IL-1 and TNF levels are significantly correlated with fatigue, sadness, autonomic symptoms, and a flu-like malaise [252]. Moreover, females diagnosed with chronic fatigue syndrome show higher plasma concentrations of IL-1α, IL-1β, IL-4, IL-5, IL-6, IL-12, and TNF-β, and lower concentrations of IL-8, IL-13, and IL-15 [123]. In support of a role for innate immune activation in unexplained fatigue and unwellness, Raison and colleagues showed in a large population-based sample that plasma concentrations of CRP, blood leukocyte numbers, and a combined inflammation factor, which benefits from the combined predictive values of both variables while minimizing measurement errors of the single components, are increased significantly or by trend in individuals with chronic fatigue syndrome and unwellness symptoms that did not meet diagnostic criteria for chronic fatigue syndrome (defined as “insufficient fatigue”) when compared to healthy controls [332]. Isolated PBMCs from individuals diagnosed with chronic fatigue syndrome/myalgic encephalomyelitis, in comparison to those from healthy individuals, further secret more IL-10, IFN-γ, and TNF when stimulated ex vivo with phytohemagglutinin, providing additional support for increased immune (re)activity in chronic fatigue syndrome [49].

### Risk factors for mental disorders promote immune hyperreactivity to psychosocial stress

From an evolutionary perspective, it makes sense that activation of the innate, rather unspecific and, therefore, fast-acting immune system has been selected to be an inevitable part of the classical stress response. Typical stressors faced by animals and non-human and human primates during evolution were mostly of a physical nature, acute duration, and comprised of conflicts among conspecifics related to hierarchy formation or exposure to various predators, both implying an increased risk of being injured and, consequently, infected by different pathogenic microorganisms [279]. Thus, individuals showing an activated immune status, even before the actual physical injury and pathogenic invasion happens, elicited by perceiving a certain situation psychologically as threatening or dangerous, had an evolutionary benefit and were selected over the past millions of years [279]. Most stressors faced by humans in the modern and developed world are exclusively psychosocial nature, lacking any component of physical injury. Thus, although there is little to no risk of being injured and colonized by pathogens as a consequence of exposure to most stressors nowadays, psychosocial stressors activate these evolutionarily conserved patterns and key inflammatory pathways [285, 378]. Although the mechanistic details underlying this kind of “sterile immune activation” will be discussed in “The role of DAMPs, MAMPs, PAMPs and the inflammasome in stress-induced “sterile” inflammation,” this response is indicated by marked increases in circulating levels of proinflammatory cytokines, such as IL-6, induced by NF-κB signaling in peripheral blood mononuclear cells (PBMCs) [36, 309, 425]. Interestingly, the latter is more pronounced in individuals at high risk for developing affective disorders [159, 187, 308], supporting the hypothesis that mental disorders are not just accompanied by chronic low-grade inflammation, as outlined in “Stress, inflammation, and mental health: The cytokine theory of affective disorders,” but at least in part also promoted by stress-induced immune activation.

#### Early-life adversity

Healthy men and women with a history of childhood maltreatment show greater overall peripheral release of IL-6 during a standard psychosocial stress challenge (the Trier Social Stress Test (TSST)), as compared with the control group [61]. Considering how often each human individual faces psychosocial challenges throughout life, it is not surprising that childhood maltreatment is an independent, but preventable risk factor for inflammation in childhood and adulthood, characterized by increased levels of proinflammatory cytokines and CRP, fibrinogen, and white blood cells [28, 75, 76, 388]. Interestingly, as non-steroidal anti-inflammatory treatment is able to prevent delayed effects of maternal separation in rats [48], it is likely that early pharmacological interventions targeting inflammation may be effective in preventing the long-term consequences of early-life adversity in humans. Independent of facing additional social stressors, spontaneous production of proinflammatory cytokines in isolated immune cells was also higher in women with a history of childhood maltreatment [40]. Three other studies further found exaggerated IL-6 responses to ex vivo stimulation of toll-like receptors 3, 4, and 5 in adolescents raised in harsh family environments [281], in adults raised in low socioeconomic status [282] and in adolescent girls with early-life adversity [107, 112]. These human data are in line with work done by our group and others, demonstrating increased immune (re-)activity [27, 401, 406], anxiety-related behavior [348, 405], and psychosocial stress vulnerability [406] in adult rodents exposed to maternal separation from postnatal days 1–14, an internationally accepted animal model for early-life stress/trauma [149, 159, 175, 184, 209, 297, 322, 360, 404, 422].

#### Low subjective social status

In addition to childhood adversity, lower subjective social status (SSS), which reflects where a person positions her- or himself on a social ladder in relation to others, goes along with exaggerated IL-6 responses to TSST exposure [95]. Interestingly, individuals who see themselves as lower in social standing are also at greater risk for poor health in general [173, 371] and for developing depression in particular [93]. Moreover, healthy young participants who were lower in self-compassion exhibit significantly greater IL-6 responses when exposed to the TSST, even when controlling for self-esteem, depressive symptoms, demographic factors, and distress [47]. In line with these findings, engagement with Cognitively Based Compassion Training (CBCT) reduced CRP from baseline to the 6-week time point after assessment in adolescents participating in a foster care program [311], suggesting that inflammatory measures relevant to health in adolescents at high risk for poor adult functioning as a result of significant early-life adversity can be reduced by increasing their self-compassion.

#### Adiposity/obesity

Moreover, individuals with higher measures of adiposity exposed to the TSST on two subsequent days showed higher IL-6 baselines on both study days, as well as sensitization of IL-6 responses to repeated acute psychosocial stressor exposure [272]. In contrast, among normal weight individuals, acute psychosocial stress induces an increase in plasma IL-6 [378], which does not typically habituate but also does not sensitize to repeated stressor exposure [345, 417]. Obesity is of epidemic proportions in the USA and in many other parts of the world and has been shown to be positively associated with various stress-associated somatic disorders, including cardiovascular and liver disease, dyslipidemias, certain forms of cancer, inflammatory diseases, stroke, and type II diabetes [199, 218, 220, 365, 366], as well as affective disorders, including depression [91, 240], in the general population.

#### Urban upbringing/living

The reader is kindly directed to “Mental disorders” and “Upbringing in areas with a wide range of microbial exposure dampens immune reactivity towards psychosocial stressors,” in which we detail that urban upbringing/living is paralleled by both an increased prevalence of mental disorders and an increased immune activation towards acute psychosocial stress induced by the TSST.

### Chronic psychosocial stress induces chronic immune activation in healthy individuals

Current knowledge on the effects of chronic psychosocial stress on chronic low-grade inflammation in humans has been reviewed in detail recently [345], with a focus on caregiver stress, work-related stress (including unemployment and burnout) chronic stress related to low socioeconomic status (SES), early-life stress induced by childhood adversity and maltreatment, and self-reported chronic stress. The conclusion drawn by the author was that current evidence is supportive of increased markers of systemic inflammation among individuals experiencing chronic psychological or social stress [345]. The most consistent evidence in this respect is coming from caregiving paradigms and studies relating early-life adversity or maltreatment to current levels of circulating inflammatory molecules [345]. In terms of caregiving stress, most studies so far focused on the effects of family dementia caregiving and consistently report elevated plasma IL-6 levels [143, 241, 265, 416], whereas increased CRP levels are found in some but not all studies [143, 416]. In contrast to Alzheimer’s caregiving, the experience of caring for a family member suffering from and being treated for glioblastoma multiforme, the most common and most aggressive primary brain tumor, resulted in a profound linear increase in systemic inflammation in the year after diagnosis, as indexed by CRP, but not IL-6. At the same time, brain tumor caregivers displayed a linear decline in mRNA for anti-inflammatory signaling molecules like nuclear factor of kappa light polypeptide gene enhancer in B-cells inhibitor, alpha (I-κBα), and diminished in vitro GC sensitivity [347]. Their monocytes showed a diminished expression of transcripts bearing GC response elements (GREs) and a heightened expression of transcripts with response elements for NF-κB, as well as a greater production of the inflammatory cytokine IL-6 during ex vivo LPS stimulation [283, 284].

In terms of early-life stress, childhood maltreatment has been shown to cause chronic low-grade inflammation, characterized by increased levels of proinflammatory cytokines and CRP, fibrinogen, and white blood cells [28, 76, 388]. Spontaneous (non-stimulated) production of proinflammatory cytokines in isolated immune cells was also higher in women with a history of childhood maltreatment [40]. Besides the immunoenhancing effects of these severe early-life stressors, also comparatively mild stressors, such as low childhood SES, indicated by socioeconomic conditions such as a lack of home ownership or low parental education, were significant predictors of inflammatory potential in adulthood, as evidenced by increased expression of inflammatory genes in circulating immune cells [280, 282] and increased plasma IL-6 and CRP concentrations [312]. In line with the latter and findings reported above for women with a history of childhood maltreatment, three other studies found exaggerated IL-6 responses to ex vivo stimulation of toll-like receptors 3, 4, and 5 in adolescents raised in harsh family environments [281], in adults raised in low socioeconomic status [282], and in adolescent girls with early-life adversity [107, 112]. Reports of severe adversity in the form of documented abuse were further associated with a 73% greater risk of first hospital treatment of asthma and more frequent asthma-related hospitalizations [144, 216]. Of particular relevance in the context of the current review is the fact that psychosocial stress activates peripheral inflammatory pathways [345, 378] and does so more robustly in people with histories of early-life abuse and/or neglect [61, 309] who are also at significantly heightened risk for PTSD development in response to trauma exposure in adult life [308].

### Human data suggesting a causal role of (stress-induced) immune activation in the development of stress-associated mental disorders

Prospective human and mechanistic animal studies (see “Animal data suggesting a causal role of stress-induced immune activation in the development of stress-associated mental disorders”) strengthen the idea that an exaggerated immune (re)activity plays a critical role in the development of mental disorders [192, 201]. For instance, individuals with inflammatory diseases are three to four times more likely to experience depression [69, 99, 103, 254, 441]. Moreover, although low-dose intravenous injection of *Salmonella abortus equi* endotoxin (0.8 ng/kg body weight) had no effects on physical sickness symptoms, blood pressure or heart rate, elevation of circulating cytokine levels (TNF, soluble TNF receptors, IL-6, IL-1 receptor antagonist) was positively correlated with endotoxin-induced anxiety levels and depressed mood and negatively correlated with verbal and non-verbal memory functions [339]. Thus, a mild stimulation of the primary host defense has negative effects on emotional and memory functions, which are probably caused by cytokine release [339]. In line with this hypothesis, a higher production of the proinflammatory cytokine IL-1β during ex vivo LPS stimulation of venous blood samples predicted a greater increase of depressive symptoms, whereas that of its natural antagonist IL-1ra predicted a smaller increase of depressive symptoms [399]. Interestingly, although a single infusion of low-dose endotoxin derived from *Escherichia coli* (0.8 ng/kg body weight) in 115 human volunteers (69 females, 46 males) led to comparable increases in the plasma concentration of the proinflammatory cytokines TNF and IL-6 in men and women, the latter showed greater increases in depressed mood and feelings of social disconnection [287], in line with data showing that women are more likely to develop mood disorders compared with men [145, 191]. Importantly, Engler and colleagues showed in healthy male volunteers that intravenous administration of low-dose endotoxin (0.8 ng/kg body weight) not only induces a significant increase in peripheral blood cytokine concentrations of TNF, IL-6, and IL-10 but also results, with some delay, in a robust and selective increase of IL-6 in the CSF [113]. The latter was strongly positively associated with the severity of mood impairment [113], suggesting that the appearance of depressive symptoms in inflammatory conditions might be primarily linked to an increase in central IL-6. The causal role of the immune system in stress-related mood disorders in general, as well as the prominent role of IL-6, is supported further by findings showing prospectively that a “low IL-6” synthesizing genotype was associated with significantly fewer symptoms of depression during IFN-α and ribavirin treatment of 98 Caucasian patients, due to chronic hepatitis C virus infection [53]. Higher levels of the systemic inflammatory marker IL-6 in childhood are associated with an increased risk of developing depression and psychosis in young adulthood [192]. Moreover, data collected within the framework of the Whitehall II cohort study further indicate that plasma IL-6 concentrations in mentally healthy participants are predictive for their likelihood of symptoms of mental disorder later in life. In detail, compared to participants with low IL-6 in 1997, those with high IL-6 had a greater likelihood of symptoms of mental disorder in 2003 and/or 2008; the prevalence of new-onset mental disorder in 2003 and/or 2008 was even higher among those who had high IL-6 in 1992, 1997, and 2003 [201].

Besides plasma IL-6, baseline CRP levels also have been shown to predict development of mental disorders. For instance, higher baseline plasma CRP levels in 267 mentally healthy mixed sex participants at the age of 85 years preceded an accelerated increase in depressive symptoms assessed by the Geriatric Depression Scale in a prospective 5-year follow-up study. Plasma CRP levels assessed in soldiers prior to war zone deployment were further predictive for development of postdeployment PTSD symptomatology, even after adjusting for differences in baseline PTSD scores, severity of trauma exposure, and other relevant covariates [114]. Moreover, genetic variability in the *CRP* gene resulting in increased serum CRP level was positively associated with PTSD symptom severity, including that of hyperarousal symptoms, exacerbated fear-related psychophysiology and PTSD symptom ratings and diagnosis [402].

The important role of particularly stress-induced immune activation in the development of mental disorders is suggested by prospective studies linking acute stress/trauma-induced immune activation with development of mood disorders later in life. For instance, morning serum IL-6 concentrations, measured in children within the first 24 h after a motor vehicle accident, were higher in children that developed PTSD 6 months later, relative to those who did not and those of the control group, and predicted PTSD development 6 months later [319]. Of particular importance in this context, psychosocial stress has been shown repeatedly to activate peripheral inflammatory pathways [345] and to do so more robustly in people with histories of early-life abuse and/or neglect [61, 378], who are also at significantly heightened risk for PTSD development in response to trauma exposure in adult life [308].

### Inflammation as a predictor of antidepressant response

Evidence suggests that inflammation may be a predictor of antidepressant response. Cattaneo and colleagues showed in depressed patients that inflammation status is a major predictor of antidepressant response. In detail, absolute measurements of MIF and IL-1β levels above a certain threshold accurately predict non-responsiveness of these patients to standard antidepressants, suggesting that it might be possible to use these cutoffs to direct certain patients towards earlier access to a combination of antidepressants and anti-inflammatory drugs [62]. In line with this hypothesis, anti-inflammatory drugs, e.g., the anti-TNF antibody, infliximab [333], or the cyclooxygenase (COX) inhibitor celecoxib [202, 292], have shown some promise in treatment of stress-related psychiatric disorders. Interestingly, and in support of the latter, acutely bereaved participants, assessed within 30 days of the death of their spouse, reported significantly fewer depressive symptoms when treated with 81 mg of aspirin per day over the preceding 5 days, compared to bereaved participants receiving placebo treatment only [185].

### Antidepressants normalize systemic cytokine levels

A recent meta-analysis further indicates that there may be a normalization of overactive inflammatory processes following standard antidepressant treatment. In detail, pooled effect sizes indicate a significant decrease in IL-6 and a less pronounced decrease in CRP after antidepressant treatment [164]. Of note, although meta-regression in this meta-analysis revealed no significant association between baseline IL-6 or CRP and change in depressive symptoms during standard antidepressant therapy, the pattern across the included studies was that higher baseline IL-6 and CRP were related to larger decreases in depressive symptoms [164]. Additionally, meta-regression showed no significant relationship between percentage of individuals who responded to treatment and inflammatory marker change; however, at the individual study level, there was evidence of decreases in IL-6 for treatment responders, but not treatment non-responders [164, 437].

### Animal data suggesting a causal role of stress-induced immune activation in the development of stress-associated mental disorders

In line with human studies, studies in laboratory rodents also clearly show that systemic or central infusion of bacterial endotoxins or proinflammatory cytokines induces “sickness behavior” reminiscent of depressive symptoms. Due to a plethora of excellent studies by Dantzer and colleagues, reviewed in detail elsewhere [58, 77–80, 82], it is also quite well-understood how this is mediated, at least in animals. A landmark paper, recently been published by Hodes and colleagues [165], provides evidence that psychosocial stress-induced inflammation is causally involved in the development of anxiety- and negative affective-related responses. In detail, they have shown that male mice responding with higher plasma IL-6 concentrations to a single acute social defeat exposure are more vulnerable to developing social deficits when exposed to social defeat repeatedly. In confirmation that these effects are due to innate differences in immune reactivity and not to differences in the severity of bite wounds received during social defeat, increased blood leukocytes in general, and monocytes in particular, as well as LPS-induced ex vivo IL-6 secretion predicted enhanced stress vulnerability in stress-naïve male mice. In confirmation of the critical role of IL-6 secreted during repeated stressor exposure in mediating stress-induced social deficits, bone marrow (BM) chimeras generated by transplanting hematopoietic progenitor cells from stress-susceptible mice releasing high IL-6, but not chimeras generated from IL-6 knockout (IL-6−/−) mice, into irradiated recipient mice, showed increased stress vulnerability when repeatedly exposed to social defeat. Moreover, anti-IL-6 antibodies prevented social deficits in vulnerable BM chimeras exposed to repeated social defeat.

## Underlying mechanisms

### The role of DAMPs, MAMPs, PAMPs, and the inflammasome in stress-induced “sterile” inflammation

This topic has been covered recently in a number of excellent reviews [122, 279]. Briefly, the innate immune system engages an array of germline-encoded pattern-recognition receptors (PRRs) to detect invariant microbial motifs and to mount a fast and unspecific innate immune response. PRRs are thus expressed by cells at the front line of defense against infection, including macrophages, monocytes, dendritic cells, neutrophils, and epithelial cells, as well as cells of the adaptive immune system [361]. For instance, the extracellular milieu and the endosomal compartment of phagocytes are scanned by membrane-bound TLRs and C-type lectins (CTLs) for pathogen- (PAMP) or commensal microbial (MAMP)-associated molecular patterns, often resulting in the activation of the NF-kB and AP-1 transcription factors that drive the production of either inactive cytokine precursors, such as pro-IL-1 and pro-IL-18 [274], or active cytokines and chemokines, such as IL-6, IL-10, and MCP-1. In addition, TLR and CTL receptor binding has been shown to activate members of the interferon regulatory transcription factor (irf) family that mediate type I IFN-dependent antiviral responses [361]. In contrast, the intracellular compartment is sensed by cytosolic nucleotide-binding oligomerization domain (NOD)-like receptors (NLRs) [361], which assemble into high-molecular weight, caspase-1-activating platforms called “inflammasomes,” which control maturation and secretion of important proinflammatory cytokines such as IL-1β and IL-18 [361, 387] after recognizing pore-forming and cell permeable soluble or phagocytosed and endosome/lysosome damaging particulate or crystalline PAMPs and MAMPs [274]. Other inflammasome-independent cytokines and chemokines, such as IL-6 and IL-10, and MCP-1, do not require posttranslational cleavage by caspase-1 [275]. Like other caspases, caspase-1 is synthesized as an inactive zymogen (pro-caspase-1) and becomes proteolytically active only after controlled dimerization in inflammasomes that are built around one of several different molecules [387]. PAMPs and MAMPs that stimulate NLRs can include bacteria-associated RNA, DNA, pore-forming toxins, and peptidoglycans [274]. Importantly, these NLRs further recognize host-derived danger-associated molecular patterns (DAMPs), suggesting that NLRs are general detectors of cellular stress resulting from sterile trauma, intrinsic metabolic disturbances, or pathogen infection [274]. Some of the host-derived DAMPs that activate NLRP3, which is the best-characterized NLR capable of forming an inflammasome, including hyaluronan, cholesterol crystals, extracellular ATP, β-amyloid, DNA, heat shock proteins (HSPs), uric acid, hyaluronan and monosodium urate crystals, high mobility group box 1 (HMGB1), and reactive oxygen species (ROS) [274, 279, 387], while environmental DAMPs include asbestos, silica, nanoparticles, skin irritants, and alum adjuvant. DAMPs can accumulate as a result of metabolic disorders or may be released upon cellular damage caused by trauma (i.e., myocardial infarction, thorax trauma, fracture) and infection, contributing to sterile inflammation and wound responses, as well as pathogen-associated immune responses [274].

Besides the directly immunomodulatory hypothalamic–pituitary–adrenal (HPA) axis and the sympathetic nervous system (SNS) [45, 96–98, 155, 156, 286, 385, 386, 413], the inflammasomes represent a crucial immunological interface between stress and inflammation [279]. Of particular importance in the context of stress-evoked sterile inflammation [122, 344] is that psychosocial stress is able to increase both DAMPs (i.e., Hsp72 and uric acid) and MAMPs [181, 260, 261], which are, as outlined above, both able to drive inflammasome activation and thus, peripheral cytokine release. Although the detailed mechanisms are not fully understood, the DAMP Hsp72 has been shown to be systemically released via a catecholaminergic, but not glucocorticoid-mediated mechanism [122, 180, 181]. A role of MAMPs in stress-induced immune activation is suggested by the finding that germ-free (GF) compared with conventionally housed mice lack the well-known social stress-induced increase in microbicidal activity and the enhanced cytokine mRNA expression in splenic macrophages when exposed to the social disruption stress (SDR) paradigm [8]. In line with this hypothesis, colonizing GF mice with a conventional microbiome rescued typical SDR-induced increases in splenic macrophage reactivity [8, 19]. Support for a causal link between stress-induced elevations of systemic MAMP levels and innate immune activation is further provided by data showing that treatment of conventionally housed mice with antibiotics attenuates both the SDR-induced increase in serum peptidoglycan levels, representing a typical MAMP, and elevated splenic macrophage reactivity [8]. In another study, exposure to SDR failed to increase plasma IL-6 and MCP-1 concentrations in antibiotic-treated mice, while these cytokine concentrations correlated with stressor-induced changes in the relative abundances of three bacterial genera (i.e., *Coprococcus*, *Pseudobutyrivibrio*, and *Dorea*) assessed in the cecum [20]. Interestingly, mice exposed to the chronic subordinate colony housing (CSC) paradigm [73, 133, 134, 334, 368], a preclinically established rodent model for PTSD [336] (for more information, see Table 1), which promotes splenocyte activation, as seen following SDR exposure [124, 335], increased also many circulating pro- and anti-inflammatory cytokines, including IL-1β, IL-6, IL-10, granulocyte colony stimulating factor (G-CSF), and MCP-1 [214]. Although we cannot delineate whether the systemic immune activation seen following CSC exposure is mediated by increased DAMPs or MAMPs, we can exclude involvement of any kind of PAMPs, as these experiments have been performed under specific pathogen-free (SPF) conditions [214, 215]. An interesting study in this context shows that both reducing commensal bacteria using antibiotics and neutralizing LPS using endotoxin inhibitor (EI) attenuate increases in some inflammasome-dependent (IL-1β and IL-18), but not inflammasome-independent (IL-6, IL-10, and MCP-1) inflammatory proteins in the blood of male F344 rats exposed to an acute tail shock stressor [261]. In this context, it is important to mention that it has been shown in vitro that administration of a MAMP or DAMP alone is not sufficient for activating the inflammasome [108, 295]. Only co-administration of both ligands is able to activate the inflammasome and start cytokine production [295]. Thus, it has been hypothesized by Maslanik and co-workers that DAMPs released during stressor exposure in vivo likely act as the first signal in stress-evoked cytokine and chemokine production, underlying the stress-induced release of the inflammasome-independent cytokines and chemokines (i.e., IL-6, IL-10, and MCP-1) [261]. Stress-associated barrier defects and elevations in systemic MAMPs likely provide the second signal necessary for upregulation of inflammasome-dependent cytokine production (i.e., IL-1β, IL-18).

**Table 1:** Comparison of patients diagnosed with posttraumatic stress disorder (PTSD; left column) and mice exposed to the chronic subordinate colony housing (CSC; right column) paradigm

PTSD	CSC
Re-experiencing of aversive details of the traumatic event(s) [12]	Re-exposure to social defeat [213]
Avoiding of trauma-related external reminders [12]	Social deficits towards unfamiliar male conspecifics (SPAT) [213]
Negative cognitions and mood [12]	Persisting anxiety (EPM, LDB, OF, OA, EPF, SPAT) [213]
Hyperarousal [12]	Increased locomotion and elevated NE [213]
Gastrointestinal pathology [43]	Development of spontaneous colitis, aggravated DSS colitis [213]
Basal hypocortisolism [85]	Basal hypocorticism [213]
Flattened cortisol rhythm [434]	Flattened corticosterone rhythm [213]
Increased DEX suppression of ACTH [433]	Increased DEX suppression of FS-induced ACTH [213]
Increased HPA axis response towards novel stressors [87]	Increased HPA axis response towards EPF [213]
Reduction in % plasma Treg cells [373, 421, 442]	Reduction in % Treg cells in peripheral lymph nodes [213]
Comorbid osteoporosis and increased fracture risk [138, 139]	Compromised bone metabolism [125] and regeneration [150]
Chronic low-grade inflammation [142, 233]	Systemic immune activation [73, 124, 214]
Comorbid alcohol abuse or dependence [119, 321]	Increased ethanol consumption [320]

*ACTH* adrenocorticotropic hormone, *DEX* dexamethasone, *DSS* dextran sulfate sodium, *EPF* elevated platform, *EPM* elevated plus-maze, *FS* forced swim, *HPA* hypothalamic–pituitary–adrenal, *LDB* light–dark box, *NE* norepinephrine, *OA* open-arm exposure, *OF* open-field, *SPAT* social preference/avoidance test

### Signaling of peripheral inflammation to the brain

We indicated above that chronic stress can potentially cause chronic inflammation [32] and that the latter in turn is causally involved in the development of many chronic stress-related mental disorders like PTSD and depression, as well as medical conditions, including burnout syndrome and chronic fatigue syndrome (see “Human data suggesting a causal role of (stress-induced) immune activation in the development of stress-associated mental disorders”). Nevertheless, given that the source of such inflammation on the one hand is predominately located at the peripheral and systemic level [32], and that the CNS on the other hand represents, in large part, an “immune-privileged” system [136, 236], protected from surrounding immunological responses, the question arises how peripheral and systemic inflammation is able to reach and to affect the brain. Intensive research in this field has shown that the communication of inflammatory signals to the brain occurs via several different pathways. These include the transmission and production of inflammatory mediators at specific regions of the blood–brain barrier (BBB; humoral route), cytokine-mediated activation of afferent nerve fibers in the periphery (neural route), and the trafficking of peripheral immune cells into CNS tissue (cellular route). These pathways have already been intensively addressed in recent review articles [79, 83, 84, 279] and are, therefore, only briefly summarized here.

#### Humoral route

The first indications that inflammatory mediators are able to functionally act within the CNS came from studies showing that centrally-injected cytokine receptor antagonists are able to influence the effects of peripherally-induced inflammation in rodents [183, 211]. Given the highly selective permeability properties of the BBB, it was long assumed that cytokines are only able to pass the BBB at the circumventricular organs (CVOs), which are brain structures that lack the impermeable characteristics of the BBB. The latter include the pineal gland, parapineal organ, vascular organ of the lamina terminalis, subfornical organ, paraventricular organ, neuropituitary, median eminence, subcommissural organ, and the area postrema and the choroid plexus [137]. In support of this theory, lesion studies in rodents indicated that a disruption of specific parts of the CVOs dampens fever, induced by peripheral LPS application [389], as well as prevents peripheral IL-1β-induced HPA axis activation [222]. Nevertheless, a direct passage of cytokines is not the most prominent way of immune-to-brain communication at the level of the CVOs. The latter seem to predominantly function as a relay station for signal transduction of inflammatory signals from the periphery to the CNS. Similar to processes taking place in the periphery (see “The role of DAMPs, MAMPs, PAMPs and the inflammasome in stress-induced “sterile” inflammation”), CVOs comprise of immunologically active cells that are able to sense PAMPs, DAMPs, or MAMPs, as well as cytokines, via the expression of TLRs [210, 328] and receptors for IL-1β [57, 115], TNF [293], IL-6 [398], and CD14 [208], respectively. Binding of these receptors in turn results in the production and release of inflammatory mediators including IL-1β [140], TNF [294], IL-6 [398], nitric oxide synthase ([426], NO-synthase), as well as the thermoregulatory [382] and proinflammatory factor PGE_2_ [33], into the perivascular compartments. Once these signaling molecules enter the brain, they can either directly interact with central non-neuronal immune cells [29, 115] to induce local inflammatory processes [329] or activate nerve terminals [383] to influence neural signaling to other brain regions [383].

In addition, independent of the CVO-mediated route of signaling, various types of cytokine receptors are also present on endothelial cells of the BBB [109, 205, 293, 398]. Given that the BBB endothelium itself can produce different types of cytokines [22], and also expresses COX-2 in response to systemic immune activation [57], which is involved in PGE_2_ synthesis and, thus, thermoregulation, it is very likely that inflammatory signals acting directly at the BBB itself also contribute to immune-to-brain signaling. In support of the latter, Banks and colleagues were able to describe the presence of another CVO-independent route, consisting of selective cytokine transport systems within the BBB [21]. Since this discovery, many cytokines, including IL-1β [23, 26], TNF [148], and IL-6 [24], have been shown to cross the BBB via specific transporters, whereas other cytokines, like IL-2 or IL-10, do not have the ability to enter the brain via this route [25, 186]. Moreover, these saturable cytokine transport systems seem to be specific to closely related cytokines and strongly differ in the rate of transport [21].

#### Neural route

Given that inflammation often occurs locally in the periphery, it cannot always be sensed by the brain via CVO- and/or BBB-mediated pathways. Thus, it is reasonable that the CNS is able to receive peripheral inflammatory information by ways different from the humoral route. In this context, Watkins and colleagues proposed that sensory inputs of peripheral afferent fibers can also transmit inflammatory information to the brain [253]. This hypothesis is supported by the finding that rat dorsal root ganglia afferent neurons express IL-1β receptors [306], and that systemically injected antibodies against IL-1β and TNF are able to reduce the fever response to peripherally-injected LPS in rodents [235]. Afferents of the vagus nerve seem to especially be important in this context, as vagal paraganglia cells are able to bind IL-1β [141], and peripheral IL-1β administration is able to activate vagal sensory neurons [110]. The latter are known to project to the nucleus of the solitary tract, which relays the information to other autonomic, as well as stress-relevant, brain regions like the paraventricular nucleus of the hypothalamus (PVN), the amygdala, and the bed nucleus of the stria terminals (BNST) [41, 42], where these sensory inputs in turn induce an adequate physiological, endocrine [41], and behavioral response [84]. These findings are supported by the fact that vagotomy prevents fever responses following peripheral injection of IL-1β [420] and behavioral responses following peripheral injection of LPS [221]. In addition, although relatively unexplored, sympathetic afferents, afferent fibers that travel within the sympathetic nerve bundles, with cell bodies in the dorsal root ganglia are also likely to contribute to relaying signals of peripheral inflammation to the CNS. These afferents have the potential to relay signals from cutaneous and mucosal surfaces, as well as viscera to the CNS via spinothalamic, spinoreticular, spinomesencephalic, spinoparabrachial, spinohypothalamic, spinocervical, spinovestibular, spinoolivary, and other spinal afferent pathways to the CNS [161, 188, 268, 269].

#### Cellular route

The cellular route of immune-to-brain communication involves the migration of activated peripheral immune cells into brain tissue. This route has recently been described by Swain and colleagues, who were able to show that hepatic inflammation [190] in mice is associated with an increased infiltration of monocytes to the brain. The mechanism behind this phenomenon has already been described. Given that brain microglial cells have shown to produce MCP-1 in response to systemic elevation of TNF, and that immune-to-brain cell trafficking seems to be abolished in the absence of MCP-1, its receptor C-C chemokine receptor type-2 (CCR-2), as well as the TNF-receptor 1 (TNFR1) [74], it is proposed that the production of brain-derived MCP-1 in response to high levels of TNF attracts peripheral leukocytes to migrate into the CNS. In turn, these immune cells are TNF-positive and are assumed to activate residing brain macrophages to produce additional TNF [190]. Of note, psychosocial stress in mice, induced by repeated social defeat, also results in an influx of monocytes into the brain [424]. In line with these findings, the latter has shown to be dependent on the presence of CCR-2 and is associated with an increase of anxiety-related behavior.

### How do inflammatory signals contribute to the development of mental disorders?

It is commonly accepted that inflammatory mediators can alter mood and behavior in part by influencing the monoaminergic neurotransmission in the brain [5, 92]. This has been referred to as the so-called monoamine deficiency theory and has been reviewed previously [279]. Briefly, the basis of this concept is that neuroinflammation causes a general depletion of the neurotransmitters norepinephrine (NE), dopamine (DA), and especially serotonin (5-hydroxytryptamine; 5-HT). This is mediated by proinflammatory cytokines, which are supposed to influence monoamine metabolism by regulating the activity of core enzymes involved in the synthesis of NE and DA, as well as 5-HT. In support of this hypothesis, cytokine-induced oxidative stress [296, 374] has been shown to promote the degradation of tetrahydrobiopterin (BH4). The latter is an important co-factor that influences the activity of rate-limiting enzymes for DA and NE (tyrosine hydroxylase; TH) as well as 5-HT (tryptophan hydroxylase; TPH) synthesis [296]. Evidence for this mechanism also comes from human data showing that treatment with IFNα is able to reduce BH4 activity and DA availability in the CSF. In line with these findings, these effects are associated with typical symptoms of depression [121].

Inflammation can also drive the depletion of 5-HT via the activation of indoleamine-2,3-dioxygenase (IDO), an enzyme that converts the 5-HT-precursor molecule tryptophan into kynurenine [249]. This hypothesis is supported by studies showing that IFNγ and TNF [163, 318, 390, 392], as well as IL-6 [197], significantly promote IDO activity. In turn, IDO activation and tryptophan depletion have profound negative effects on mood. This is underlined by the fact that tryptophan depletion, represented by lower plasma tryptophan concentration, has repeatedly been reported in individuals with depression [250, 400], and that an increased serum kynurenine to tryptophan ratio predicts depressive symptoms in humans [111]. Moreover, IDO mRNA expression is increased in animals showing sickness behavior, and IDO-deficient mice do not show depressive-like behavior in response to bacterial infection [302]. Of note, proinflammatory cytokines also have been shown to affect the reuptake of monoamines [59]. For example, in vitro experiments indicate that TNF and IL-1β [443] increase the activation of the 5-HT transporter (SERT). This has been shown to result in a reduced synaptic abundance of 5-HT, which, in turn, is associated with depressive-like behavior in mice [444]*.*

Besides alterations in monoamine availability, an increased synaptic abundance of glutamate (Glu) has also been associated with both an increased central inflammatory status and the development of mood disorders [359]. This is indicated by findings showing that depressive, compared to healthy, individuals have higher plasma levels of this essential neurotransmitter. This effect can be diminished by antidepressant medication [207]. Accordingly, glutamatergic neurotransmission can be affected by central inflammatory processes. For example, cytokines reduce the synaptic reuptake of Glu [38] as well as increase the release of Glu [408] in astrocytes. Of note, there is also evidence that kynurenine accumulation, as a result of cytokine-induced tryptophan depletion, contributes to dysregulation of glutamatergic signaling. The latter is indicated by the facts that kynurenine is metabolized into the neurotoxic metabolite quinolinic acid (QUIN) by locally activated microglia cells [249] and that QUIN is a potent glutamate receptor (NMDAR) agonist with the ability to induce a pronounced glutamate release, resulting in excitotoxicity, which itself further promotes central inflammatory processes [86, 246].

### The microbiome–gut–brain axis

According to recent findings, the ratio of bacterial to eukaryotic cells in the human body is approximately 1:1 [363], resulting in an overall microbial mass comparable to the weight of the human brain [379, 380]. It is, therefore, not surprising that the microbial composition of the gut, which comprises the majority of commensal microorganisms [363], is thought to influence the immunology, physiology, and behavior of the host organism. In line with this hypothesis, an increasing number of studies have shown that host–microbiome communication occurs in a bidirectional manner using a number of different signaling mechanisms [70]. These include the production of neuroactive microbial metabolites and short-chain fatty acids (SCFAs), vagus-to-brain communication, tryptophan metabolism, and immune system activation [70]. Many previous studies and reviews have already focused on these specific mechanisms of gut–brain communication [60, 70, 101, 127, 128, 380]; however, it is the purpose of this review to focus on the effects of the microbiome on the inflammatory status of the host.

The mammalian immune system has adapted to tolerate commensal bacteria and to distinguish them from potentially pathogenic organisms [391]. A healthy gut microbial composition protects from the invasion of pathogens but also contributes to the regulation of immune system activity [239, 353]. For example, GF mice display a pronounced downregulation of genes related to immune activity [381] and poor epithelial barrier function [169]. But how are commensal bacteria able to communicate with the host immune system? Microbes in large part mediate their immunomodulatory effects by influencing the activity and maturation of immune cells [429]. Rodent studies have provided evidence that colonizing GF mice with commensal bacteria resulted in the induction of Treg cells and the production of IL-10 [243]. Interestingly, colonization with a single bacterial strain is sufficient to increase Treg expression to levels observed in specific pathogen-free mice [307, 362]. Similarly, treatment with the probiotic bacterium *Bifidobacterium infantis* promotes the proliferation of Treg cells in mice [304] and results in an increased production of IL-10 in humans [203]. In contrast, colonization with segmented filamentous bacteria (SFB) increases the abundance of Th17 cells that express IL-17 and IL-22 [178], whereas *Bacteroides fragilis* has shown to promote Th1 responses in rodents, respectively [270]. Of note, gut microbes can also promote immune cell proliferation by stimulating cytokine production in intestinal epithelial cells. The latter is indicated by the finding that exposure to mouse cecal content in vitro induces epithelial cells to produce TGF-β [16], which is known to promote Treg induction [171].

Although the detailed underlying mechanisms are still unknown, an accumulating number of studies indicate that commensal microbes prime the host immune system via the presentation of MAMPs, like bacterial polysaccharides, which are recognized via TLR-signaling by host immune cells. Polysaccharide A, for instance, promotes T cell expansion via TLR2 activation [63, 270] and is associated with the development of IL-10-producing Treg cells in mice colonized with *Bacteroides fragilis* [63, 271]. In line with these findings, daily oral administration of the probiotic *Bifidobacterium breve* for 3 months has been shown to have similar effects in rodents [63, 179]. However, besides MAMPs, microbial metabolites like SCFA (e.g., acetate, propionate, and butyrate [410]), as well as bile acids or choline, significantly influence host immune activity. In vitro studies, for instance, indicate that butyrate not only directly facilitates the differentiation of naive CD4+ T cells into Treg cells [15] but also indirectly promotes the proliferation of Treg cells by stimulating IL-10 production by dendritic cells and macrophages [369]. Moreover, butyrate and other SCFAs promote Treg proliferation by inducing TGF-β production from gut epithelial cells [16]. Butyrate, acetate, and propionate also have been shown to reduce the expression of LPS-stimulated TNF in human neutrophil cells in vitro, as well as to reduce the expression of IL-6 in mouse colon organo-cultures [393]. In addition, bile acids not only promote innate host defense by the expression of antimicrobial genes [176] but are also able to directly inhibit bacterial overgrowth within the intestinal tract [166]. The health-promoting essential nutrient choline has been shown to have anti-nociceptive effects, reduce TNF expression in a mouse model for postoperative pain [354], and is able to modulate immune function in neonatal rats [227].

Commensal bacteria further play an important role in the production and metabolism of gut-derived tryptophan and 5-HT, which have both been implicated in the regulation of healthy innate and adaptive immune responses [18, 256]. Because of that, tryptophan metabolism has also been suggested to significantly contribute to microbial–host immune communication. For example, evidence for the latter comes from studies showing that GF mice display increased systemic levels of tryptophan and highly reduced 5-HT levels when compared to conventionally housed mice [423]. Moreover, colonizing GF mice with commensal bacteria results in significantly decreased tryptophan levels in the blood [64]. Mechanistically, microbiota can directly modify tryptophan metabolism via de novo synthesis of 5-HT [394]. The microbiota can also metabolize tryptophan into a number of bacterially derived indole metabolites that act at the aryl hydrocarbon receptor, resulting in immunoregulation and protection of the mucosa from damage [6, 217, 238]. Furthermore, microbial-derived SCFA is able to indirectly promote the production of 5-HT from epithelial cells in the gut [340].

All of the abovementioned findings suggest that a healthy commensal microbiome is an important determinant of proper host immune function. Accordingly, alterations of the gut microbial composition can have detrimental effects on host immunity. Besides factors like diet [370], age [255], and pharmaceuticals [212], one highly potent disruptor of the intestinal bacterial community is chronic stress. This is indicated by rodent studies showing that early-life stressors like the maternal separation paradigm [305], and chronic stressors in adulthood like the CSC and SDR models, result in pronounced alterations of the intestinal microbiota, as well as an increased vulnerability for inflammation [20, 215, 337, 406]. Moreover, human studies have found that various stress-related inflammatory disorders like IBD [89] and PTSD [160] are associated with changes in the composition of the gut microbiome. The inflammatory and disease-promoting potential of an unhealthy intestinal microbiome is further elucidated by studies showing that specific symptoms associated with inflammatory and stress-related disorders can be transmitted employing fecal transplantation (FT). More specifically, colonizing GF mice with the gut microbiota of IBD patients results in an increase in barrier dysfunction and innate immune activation in recipient mice [90]. Furthermore, oral FT from depressed patients is able induce depressive-like behavior in rats pretreated with antibiotics [189]. In line with these findings, colonizing GF mice with the microbiome of another mouse strain via oral gavage results in the transfer of strain-specific behavioral characteristics including exploratory behavior [31, 67].

### Why do some individuals show hyperinflammation: the role of Treg cells

Given that PTSD [373] and depression [230] are associated with decreased CD4^+^CD25^+^FoxP3^+^ Treg cell numbers, one hypothesis is that a failure of immunoregulation promotes an over-reacting of the inflammatory response to trauma or stressful life events, and thus, predisposes those individuals to the development of stress-related disorders in general, and PTSD and depression in particular. In line with the hypothesis that a compromised Treg cell compartment might be critically involved in the promotion of a dysregulated and chronically activated inflammatory immune response, and in turn, the development of stress-associated disorders, the tricyclic antidepressant, desipramine, increases the number of Treg cells in a mouse model of allergic rhinitis (AR). The latter is characterized by sneezing, nasal scratching, and increased numbers of eosinophils in the nasal mucosa, ovalbumin (OVA)-specific IgE serum antibodies, increased concentrations of IFN-γ and IL-4 in the nasal lavage fluid, and IL-17^+^ splenocytes, while splenic Treg cells are reduced [441]. Patients suffering from AR have a higher incidence of anxiety, depression, and sleep disorders than the general population [99, 441] and, in severe cases of AR, are even at higher risk for committing suicide [325]. The importance of antidepressant therapy in AR has been increasingly recognized [99, 441]. Evidence for an immunoregulatory role of Treg cells and for a dysregulated and overshooting immune system in individuals with a compromised Treg cell function is provided both by human and animal studies. For instance, *Foxp3* mutant mice develop an intense multiorgan inflammatory response associated with allergic airway inflammation, hyperimmunoglobulinemia E, eosinophilia, and dysregulated Th1 and Th2 cytokine production in the absence of overt Th2 skewing [232]. Importantly, *Foxp3* mutations also underlie a homologous autoimmune lymphoproliferative disorder in human subjects, termed immune dysregulation polyendocrinopathy enteropathy–X-linked syndrome (IPEX) or X-linked autoimmunity–allergic dysregulation syndrome [30, 232]. In line with these data, a defective suppressor function of human CD4+CD25+ Treg cells has been reported in autoimmune polyglandular syndrome (APS) type II, characterized by multiple endocrine diseases initiated by an autoimmune process in the same patient [206].

## Immunoregulatory approaches to promote stress/trauma resilience

### The rationale behind immunoregulatory approaches to promote stress/trauma resilience

If immunodysregulation and subsequent chronic low-grade inflammation are risk factors for development of stress-related psychiatric disorders, including PTSD, pretreatment with an immunoregulatory agent would be expected to be protective. In line with this hypothesis, anti-inflammatory drugs, e.g., the anti-TNF antibody, infliximab [333], or the cyclooxygenase inhibitor celecoxib [202, 292], have shown some promise in treatment of stress-related psychiatric disorders. Recent findings also suggest that inflammation status is a major predictor of antidepressant response, with high levels of inflammatory biomarkers predisposing to non-responsiveness to standard anti-depressants [62]. However, anti-inflammatory approaches to treatment of stress-related psychiatric disorders are inherently limited due to the complexity of the inflammatory response, which involves many diverse mediators and signaling cascades. A more effective approach would be to activate the body’s own immunoregulatory mechanisms, which are able to comprehensively suppress unnecessary inflammation mediated by diverse signaling pathways. This strategy has the added benefit of being targeted and potentially being long-lasting, as activation of the body’s own immunoregulatory mechanisms can persist for weeks or months, and under certain conditions [352]. The half-life of newly differentiated Treg cells in mice is estimated to be 27 days [94].

### The Old Friends hypothesis/the hygiene hypothesis/the “missing microbes” hypothesis/the biodiversity hypothesis

Increased inflammation in urban environments may be due to impaired immunoregulation, which is thought to be at least partially dependent on reduced exposure (especially during early life [242]), to microorganisms with which mammals co-evolved, as has been proposed by the biodiversity hypothesis [153], missing microbes hypothesis [39], or Old Friends hypothesis [238, 351], which all have been evoked to explain the epidemic of inflammatory disease in urban environments. Throughout human evolution, the interactions between these ancestral microbiota and the innate immune system promoted immunoregulation, as they were either part of host physiology (human microbiota), were harmless but inevitably contaminating air, food, and water (environmental microbiota), or were causing severe tissue damage when attacked by the host immune system (e.g., helminthic parasites) [39, 351]. However, microbial biodiversity and overall contact with environmental and commensal microorganisms that were present during mammalian evolution and that play a role in setting up regulatory immune pathways are progressively diminishing in high-income countries, particularly in urban areas. The latter is due to sanitation, drinking water treatment, excessive use of antibiotics, changes in diet, feeding of formula milk as a replacement for breast milk, increased cesarean section birth rates, and increased time spent within the built environment [39, 257, 351, 376]. Of particular interest in this context is a recent study showing increased innate immune system activation in Hutterite compared with Amish farm children, and an ameliorating effect of dust extracts from Amish, but not Hutterite, homes on airway hyper-reactivity and eosinophilia in a mouse model of allergic asthma [377]. Living on single-family dairy farms with regular contact with farm animals in Amish farm children further goes along with a four and six times lower asthma and allergic sensitization prevalence, respectively, compared to living on highly industrialized farms with little contact with farm animals in Hutterite farm children [377]. In accordance with this hypothesis, early exposure to both pets and farm animals is able to reduce the risk of childhood asthma and other inflammatory disorders [117, 290]. Immigrant studies further suggest that differential contact with Old Friends, particularly during early life, accounts for differences in the prevalence of psychiatric disorders in rural versus urban environments [351, 352].

### Mechanisms underlying induction of Treg by Old Friends

The induction of Treg by Old Friends undoubtedly involves diverse, and in some cases, redundant and parallel, mechanisms. Although not an exhaustive list, some of these potential mechanisms are outlined below.

#### Tryptophan and bacterially and host-derived tryptophan metabolites

As mentioned above, bacterially derived tryptophan and diverse tryptophan metabolites can have immunoregulatory effects leading to induction of Treg. For example, the tryptophan metabolite melatonin induces Treg via actions on melatonin receptor 1 (MT1) [118]. Probiotic species, such as *Lactobacillus* spp., are capable of tryptophan biosynthesis and metabolism, and they generate tryptophan metabolites that activate the aryl hydrocarbon receptor (Ahr), resulting in immunoregulation [438]. Immunoregulatory bacterially-derived tryptophan metabolites that function as Ahr agonists include tryptamine, indole-3-acetaldehyde, indole-3-acetic acid, indole-3-aldehyde, and kynurenine [438]. Other bacterially derived tryptophan metabolites that interact with Ahr include indole, 3-methyl-indole, indoxyl sulfate, 6-formylindolo[3,2b]carbazole, and kynurenic acid [440]. Activation of Ahr can induce functional Treg cells that suppress inflammation [276, 330]. Specifically, Ahr induces RORγt+ Tregs [327], consistent with studies discussed above demonstrating that specific species within the gut microbiota can induce RORγt+ Tregs and mucosal immune tolerance [307, 362]. Thus, the microbiota, acting via synthesis of tryptophan and generation of tryptophan metabolites that interact with Ahr, regulates Treg differentiation in a ligand-specific manner. Besides these Treg-promoting effects of IDO-generated tryptophan metabolites, there is evidence supporting a direct role of IDO expression in DCs to promote development of a regulatory DC phenotype. In detail, co-culture of bone marrow-derived DC with stem cells isolated from adipose tissue (ASC) suppressed DC maturation, as evidenced by low expressions of CD80, CD86, and MHC-II. Moreover, ASC-treated mature DCs showed higher levels of expression of TGF-β1, IL-10, and IDO and generated a significantly higher percentage of Treg when co-cultured with naïve CD4+ T cells. Interestingly, the IDO level in ASC-treated mDCs and Treg induction effects was blocked by the ASCs pretreated with TGF-β1 siRNAs, but not IL-10 siRNAs [419].

Although these immunoregulatory and thus, stress-protective effects of IDO activation at first glance are in contrast to the above described effects of IDO activation on brain tryptophan and/or 5-HT depletion and induction of sickness behavior (see “How do inflammatory signals contribute to the development of mental disorders?”) , we hypothesize that the latter, at least in the acute context, also represents a positive adaptation increasing an individual’s regenerative capacities during stress/inflammation. Accordingly, it has recently been shown that DC expression of IDO represents a potential mechanism to terminate immune responses [154]. Moreover, chronic or long-lasting sickness behavior is generally interpreted as a negative/pathological health consequence of stress and would be counteracted by IDO-mediated Treg induction. In line with the latter, recent data from Laumet and co-workers show that resolution of inflammation-induced depression is an active process requiring T lymphocytes acting via an IL-10-dependent pathway [219].

#### SCFAs

Short-chain fatty acids (SCFAs), including acetate and propionate, can directly induce colonic Tregs and their function via activation of G protein-coupled receptor (GPCR) 43, encoded by the free fatty acid receptor 2 gene (Ffar2) [15, 372]. In addition, butyrate acts on DCs to induce Treg through inhibition of histone deacetylase (HDAC) and may induce epigenetic changes [439].

#### Microbial antigens from Old Friends that induce immunoregulatory responses

Intracellular and cell surface bacterial antigens can induce immunoregulatory responses, for example, through interaction with the PRR dendritic cell-specific intercellular adhesion molecule-3-grabbing non-integrin (DC-SIGN). Antigen interaction with DC-SIGN can interfere with toll-like receptor-mediated inflammatory responses, resulting in decreases in NF-κB signaling and decreases in proinflammatory cytokine secretion (i.e., IL-6, TNF, and IL-12), in concert with increases in IL-10 secretion [407]. Activation of DC-SIGN in DCs, perhaps involving interactions with TLR-2 [204], may be a common mechanism through which bacterial antigens derived from the Old Friends induce regulatory DCs, which can bias T cell differentiation towards a Treg phenotype (for review of specific antigens derived from Old Friends that can activate DC-SIGN, see [238]).

### Urban versus rural prevalence differences in stress-associated disorders

#### Somatic disorders

More than 50% of the world’s population currently lives in urban areas, projected to rise to 70% by 2050, with 50% of the urban population living in cities with more than 500,000 residents [396]. Although a lower prevalence of allergies in rural compared with urban Mongolia has been reported [299], the prevalence of somatic inflammatory disorders seems to critically depend rather on the extent of overall contact to microbial products during early years of development than on the rural–urban environment per se. In other words, a subject’s exposure to environmental microbial antigens seems to have a crucial role in the development of tolerance to ubiquitous allergens found in natural environments [46]. In accordance with this hypothesis, endotoxin levels in samples of dust from the child’s mattress were inversely related to the occurrence of hay fever, atopic asthma, and atopic sensitization [46], an effect that was independent from whether 6–9-year-old children grew up in farming or non-farming households within rural areas of Germany, Austria, or Switzerland. Of note, cytokine production by leukocytes was also inversely related to the endotoxin level in the bedding, indicating a marked downregulation of immune responses in exposed children. An important role for regular animal contact in increasing the overall exposure to environmental antigens and, thus, in promoting immunoregulation and protection against inflammatory somatic disorders is suggested by several studies. For instance, exposure of children to stables and consumption of farm milk was associated with lower frequencies of asthma, hay fever, and atopic sensitization [341], an effect that was more pronounced if exposure happened during the first year of life than during years 1–5. However, continuous long-term exposure until the age 5 years was associated with the lowest frequencies of these inflammatory disorders. As outlined in detail above, living on single-family dairy farms with regular contact with farm animals in Amish farm children further goes along with four and six times lower asthma and allergic sensitization prevalence, respectively, compared to living on highly industrialized farms with little contact with farm animals in Hutterite farm children [377]. In accordance with these data, early exposure to both pets and farm animals is able to reduce the risk of childhood asthma and other inflammatory disorders [117, 290]. A protective role of environments that afford a wide range of microbial exposures, such as traditional farms, is further supported by the PARSIFAL and the GABRIELA studies [106]. In both studies, children who lived on farms had a lower prevalence of asthma and atopy and were exposed to a greater variety of environmental microorganisms than the children in the reference group. In turn, diversity of microbial exposure was inversely related to the risk of asthma and the presence of certain more circumscribed exposures was also inversely related to the risk of asthma. Interestingly, similar effects have been found in studies investigating the association between living environment (urban vs. rural) and the development of allergies in pet dogs. Hakanen and colleagues, for instance, showed that, similar to dog owners, dogs also have a higher prevalence of allergies when living in an overall urban environment [151]. In line, Lehtimäki et al. found that both canine skin microbiota and the risk to develop allergies are decisively shaped by the animal’s living environment and lifestyle [224].

#### Mental disorders

Psychiatric disorders are more prevalent in urban versus rural areas [315, 317, 349, 403]. For instance, many studies have demonstrated that an urban birth or upbringing increases schizophrenia risk [228, 289, 316]. Interestingly, Pedersen and Mortensen in 2001 showed in a population-based cohort study of 1.89 million people that both the degree of urbanization at birth and during upbringing significantly increased the risk of schizophrenia [315]. A meta-analysis of urban–rural differences in prevalence of psychiatric disorders, conducted using data taken from 20 population survey studies published since 1985, revealed further that pooled total prevalence rates for psychiatric disorders were found to be significantly higher in urban areas compared with rural areas [317]. Specific pooled rates for mood disorders and anxiety disorders were also significantly higher in urban areas, while rates for substance use disorders did not show a difference. Adjustment for confounders had only limited impact on urban–rural odds ratios, which show that urban–rural differences in prevalence rates are only partly explained by population characteristics. In line with these findings, a recent large population-based cohort study of everyone born in Denmark between 1955 and 2006 revealed that people born in the capital had a higher incidence for all psychiatric disorders, except intellectual disability (ICD-10 “mental retardation”) and behavioral and emotional disorders with onset in childhood, than people born in rural areas [403]. Thus, birth in an urban environment is associated with an increased risk for mental illness in general and for a broad range of specific psychiatric disorders. Importantly, in contrast to inflammatory somatic disorders, less research has been conducted to understand the mechanisms underlying this increased prevalence of stress-associated psychiatric disorders in urban versus rural areas.

### Upbringing in areas with a wide range of microbial exposure dampens immune reactivity towards psychosocial stressors

**Fig. 1:**
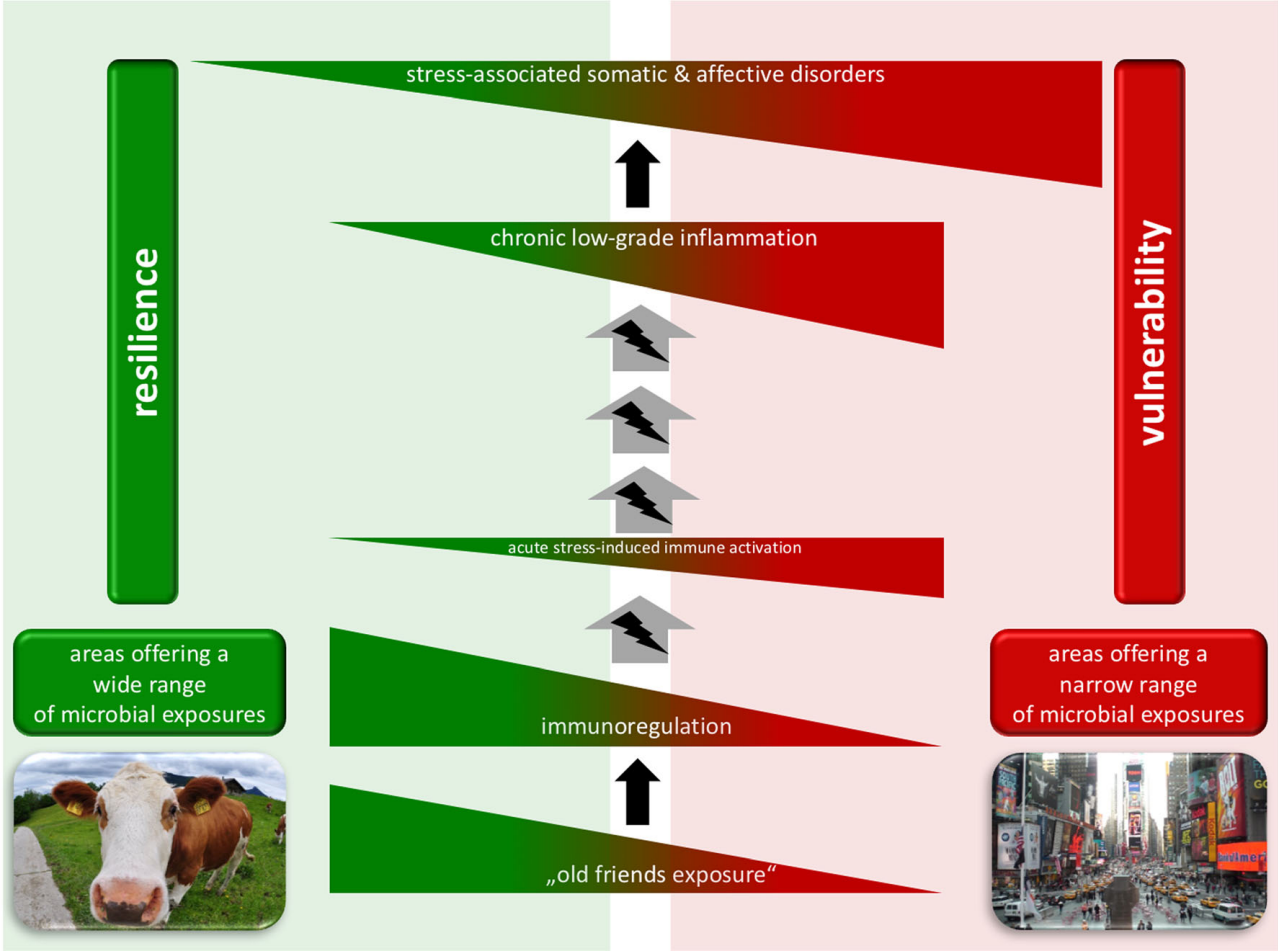
Hypothetical model illustrating how areas offering a narrow (right panel) relative to a wide (left panel) range of microbial exposures promote stress vulnerability and compromise stress resilience. Reduced exposure to immunoregulatory Old Friends, especially during early life, result in an exaggerated and long-lasting immune response towards any acute psychosocial stressor (indicated by the flash symbol in the gray arrow) faced during adulthood, over time resulting in constant immune activation and chronic low-grade inflammation and, consequently, in the development of a variety of stress-associated somatic and psychiatric disorders in which chronic, low-level inflammation is a risk factor. (Photograph on left side © Xaver Linder)

As stress-associated somatic and psychiatric disorders are more prevalent in urban compared with rural areas, or, more accurately, in environments offering a narrow compared with a wide range of microbial exposures (see “Urban versus rural prevalence differences in stress-associated disorders”), and as individuals at high risk for mental disorders show an exaggerated inflammatory response towards psychosocial stressors (see “Risk factors for mental disorders promote immune hyperreactivity to psychosocial stress”), it is likely that upbringing in environments offering a narrow range of microbial contact facilitates immune reactivity towards psychosocial stress, relative to upbringing in environments offering a wide range of microbial exposure (hypothesis summarized in Fig. 1). To test this hypothesis, we recruited young, physically, and emotionally healthy male participants, raised during the first 15 years of life either in a city with more than 100,000 residents and in the absence of pets (urban) or on a farm keeping farm animals (rural) and exposed them individually to the TSST [198]. Pets were excluded for urban participants as they potently reduce the risk for inflammatory disorders [117], likely by facilitating contact with Old Friends. As predicted, we showed an increased systemic immune activation in response to a standardized laboratory social stressor in healthy participants with an urban upbringing in the absence of pets, relative to healthy participants with a rural upbringing in the presence of farm animals, even though questionnaires, plasma cortisol, and salivary alpha-amylase indicated that the experimental protocol was more stressful and anxiogenic for the latter. In detail, urban upbringing in the absence of pets, relative to rural upbringing in the presence of farm animals, was associated with a more pronounced increase in the number of PBMCs and plasma IL-6 concentrations following acute psychosocial stress induced by the TSST. Moreover, ex vivo cultured PBMCs from urban participants raised in the absence of animals secreted more IL-6 in response to the T cell-specific mitogen concanavalin A (ConA). In turn, anti-inflammatory IL-10 secretion was suppressed following TSST in urban participants raised in the absence of animals, suggesting immunoregulatory deficits, relative to rural participants raised in the presence of animals. As we did not include participants raised in urban areas in the presence of animals and in rural areas in the absence of animals, we cannot answer the question whether the differences in stress-induced immune activation are due to urban versus rural upbringing per se, or, and according to Stein and colleagues, this is more likely [377] due to the absence versus presence of regular animal contact.

### Using Old Friends to ameliorate inflammatory somatic disorders

Immunoregulatory Old Friends have shown promise for improving outcomes in a number of models of inflammatory somatic disorders, including allergic asthma and autoimmune disorders (including IBD and type 1 diabetes) (for review, see [350]). For example, subcutaneous, intranasal, and intragastric administrations of *Mycobacterium vaccae* (*M. vaccae*) have been reported to attenuate allergic airway inflammation in mice [170, 174, 445, 446]. Furthermore, these effects have also been noted to pass down from the mother (after *M. vaccae* exposure during pregnancy) to the offspring [3, 4]. Sensitized mice born from mothers exposed to *M. vaccae* responded with decreases in IL-5 secretion (associated with allergic disease) relative to control mice after allergen airway challenge [4]. In addition to the effects of immunoregulatory Old Friends, the effects of prebiotics and probiotics on Treg induction and implications for prevention and treatment of inflammatory disease have been reviewed recently [105].

### Using Old Friends to promote stress/trauma resilience

In addition to the protective effects of various Old Friends against a plethora of inflammatory somatic disorders, Old Friends have been further shown to positively affect mood, stress coping, and fear extinction, as well as to prevent the negative consequences of chronic psychosocial stress in humans and/or rodents. In detail, repeated intradermal administration of a heat-killed preparation of *M. vaccae* (NCTC 11659), when added to standard cancer chemotherapy, significantly improved patients’ quality of life without affecting overall survival times in a non-placebo controlled trial [300, 301]. Patients in the chemotherapy-alone group had greater deterioration in their Global Health Status score than patients in the chemotherapy plus *M. vaccae* group. Moreover, our own studies show that repeated subcutaneous (s.c.) preimmunization with heat-killed *M. vaccae* activates a specific subset of serotonergic neurons in the interfascicular part of the dorsal raphe nucleus (DRI) of mice, which is associated with increases in 5-HT metabolism within the ventromedial prefrontal cortex and a shift towards proactive stress coping in the forced swim test [237, 367]. This suggests that an immune-responsive subpopulation of serotonergic neurons in the DRI is likely to play an important role in facilitating active stress coping. In support of this hypothesis, mesolimbocortical serotonergic systems, particularly those in the medial prefrontal cortex where we observed effects of *M. vaccae* on serotonergic metabolism, are thought to play an important role in regulation of coping responses and behavioral responses to uncontrollable stress [11]. Consequently, dysregulation of DRI serotonergic systems may contribute to the dysregulation of coping mechanisms in some stress-related neuropsychiatric disorders, including major depression. A shift towards proactive stress coping was further found recently by our group following repeated s.c administration of a heat-killed preparation of *M. vaccae* in a mouse model of PSTD [337], indicated by decreased submissive behavioral displays, as well as flight and avoiding behaviors, during an initial encounter with a dominant male aggressor. To induce this PTSD-like phenotype, the CSC paradigm, which is based on the repeated psychosocial traumatization (=social defeat) in combination with chronic subordination of four male CSC mice towards a dominant resident male conspecific, was used [336]. Briefly, compared with single-housed controls (SHC), CSC mice avoid trauma-related external reminders, indicated by a lack of social preference towards unfamiliar male mice, and develop a long-lasting increase in general anxiety-related behavior and alcohol consumption/preference, hyperactivity, spontaneous colitis, and an aggravated dextran sulfate sodium (DSS)-induced colitis. CSC exposure is further associated with basal hypocorticism, increased dexamethasone suppression of ACTH, increased HPA axis reactivity towards novel stressors, and reduced numbers of Treg cells, likely contributing to the overall increased inflammatory state [336]. Importantly, the above reported *M. vaccae*-induced shift towards proactive stress coping was paralleled by preventive/ameliorating effects on development of anxiety, social anxiety, spontaneous colitis, and aggravation of DSS-induced colitis in a mouse model of PTSD [337]. As shown before using a mouse model of airway inflammation [446], *M. vaccae* propagated its immunoregulatory and, thus, PTSD-protective effects via induction of Treg cells and IL-10 secretion [337]. The latter was indicated by the fact that pretreatment with an anti-CD25 antibody, but not pretreatment with a control-antibody, prevented the stress-protective effects of prior *M. vaccae* immunization. Just recently, we showed that repeated immunization with heat-killed *M. vaccae* also enhances between-session and within-session fear extinction, but not baseline acoustic startle responses and fear acquisition or expression, in the fear-potentiated startle (FPS) paradigm in rats, relative to vehicle-immunized controls [129]. Baseline acoustic startle is a sensitive measure of generalized anxiety or fear expression [356], which was also not affected by *M. vaccae* in the PTSD mouse study [337]; only psychosocial traumatization [337] and FPS training-induced [129] anxiety were ameliorated by prior *M. vaccae* administration. The facilitating effects of *M. vaccae* on fear extinction are of particular importance, as trauma-related anxiety and affective disorders, including PTSD, are characterized as persistent re-experiencing of the trauma after a traumatic experience. Thus, immunization with *M. vaccae* may be beneficial in extinction therapies (i.e., exposure therapy) that are used for reducing fear-related psychopathologies and may reduce the amount of time before beneficial effects of therapy are seen. Finally, recent studies using *M. vaccae* have shown that the same immunization protocol shifts the brain towards an anti-inflammatory phenotype, increasing IL-4 mRNA and protein expression, and upregulating genes involved in maintaining microglia in a quiescent state, such as *Cd200r1* and *Mrc1* [126, 130]. Immunization with *M. vaccae* was found to prevent stress-induced microglial priming and stress-induced exaggeration of anxiety-like behavior in a model of learned helplessness [130] and cognitive deficits in a model of postoperative cognitive dysfunction [126]. Together, these studies suggest that immunization strategies have potential to prevent negative outcomes associated with stress-induced exaggeration of inflammation and neuroinflammation.

## Conclusions

Together, the findings reported in this review article may have implications for the practice of medicine, both from the perspectives of prevention and therapeutics. For example, it has been suggested that urban societies have a dearth of microbial immunoregulatory inputs during early life [351, 352]. This is driven not only by lack of exposure to natural environments, animals, and, thus, to environmental microbes, but also by eradication of important commensals with immunoregulatory properties. For example, humans co-evolved with *Helicobacter pylori* for tens of thousands of years [257]. *H. pylori* is immunoregulatory and may confer protection against allergies, asthma, and inflammatory bowel diseases [14] but has largely been eradicated from urban populations within the last 40–50 years [326]. Our data suggest that there may be a need to replace some of these lost microbial immunoregulatory inputs. It remains to be determined if this will be best accomplished by addition of microbial immunoregulatory inputs to the diet as nutritional supplements, by immunization, and/or by increasing contact of especially young children to environmental microbes. However, there is a lack of adequate empirical data concerning all these questions in humans.
